# Are rates of clinical interventions during pregnancy and childbirth different for refugees and asylum seekers in high-income countries? A scoping review

**DOI:** 10.1186/s12884-024-06893-2

**Published:** 2024-11-12

**Authors:** Alix Bukkfalvi-Cadotte, Gargi Naha, Ashra Khanom, Amy Brown, Helen Snooks

**Affiliations:** 1 Medical School, Swansea University, Swansea, SA2 8PP UK; 2 School of Health and Social Care, Swansea University, Swansea, SA2 8PP UK

**Keywords:** Refugee, Asylum, Migrant, Intrapartum, Childbirth, Intervention

## Abstract

**Background:**

Adequate maternity care and appropriate clinical interventions during labour and delivery can reduce adverse perinatal outcomes, but unnecessary interventions may cause harm. While studies have shown that refugees and asylum seekers face important barriers when accessing maternity care, there is a lack of high-quality quantitative data on perinatal health interventions, such as induction of labour or caesarean sections, among refugees and asylum seekers and the findings reported in the literature tend to be inconsistent. Our goal was to examine and synthesise the evidence regarding the rates of intrapartum clinical interventions in women who are refugees and asylum seekers in high-income countries compared to other population groups.

**Methods:**

We conducted a scoping review of peer-reviewed studies published in English since 2011 that report original quantitative findings regarding intrapartum clinical interventions among refugees and asylum seekers in high-income countries compared to those in non refugee, non asylum seeker populations. We examined reported rates of clinical pain relief, labour induction and augmentation, episiotomies, instrumental deliveries, and caesarean sections.

**Results:**

Twenty-five papers were included in the review. Findings indicate that refugees and asylum seekers were less likely to receive pain relief, with 16 out of 20 data points showing unadjusted ORs ranging from 0.20 (CI: 0.10–0.60) to 0.96 (CI: 0.70–1.32). Similarly, findings indicate lower odds of instrumental delivery among refugees and asylum seekers with 14 of 21 data points showing unadjusted ORs between 0.25 (CI: 0.15–0.39) and 0.78 (CI: 0.47–1.30); the remaining papers report no statistically significant difference between groups. There was no discernable trend in rates of labour induction and episiotomies across studies.

**Conclusions:**

The studies included in this review suggest that asylum seekers and refugees are less likely to receive clinical pain relief and experience instrumental delivery than non-refugee groups in high-income countries. This review strengthens our understanding of the links between immigration status and maternity care, ultimately informing policy and practice to improve perinatal health and the provision of care for all.

## Background

By the end of 2022, there were over 100 million people forcibly displaced across the world, including refugees, asylum seekers, internally displaced persons and other people needing international protection [1]. Ensuring access to high-quality healthcare services for refugees and migrants is essential to promote their health and wellbeing and to reduce worldwide health inequities [2]. Research has shown that migrants can face multiple obstacles in accessing healthcare, including discrimination, poor communication, and language and cultural barriers [3–5]. Refugees and asylum seekers, who have been forced to migrate due to fear of persecution or violation of their human rights, often face additional challenges related to limited resources and lack of social support [3, 6]. Among refugees and asylum seekers, women can present particularly complex profiles, including past experiences of gender-based violence, sexual violence, and trafficking [7].

In addition to general healthcare, adequate maternity care is essential to protect the health and wellbeing of mothers and infants [8], but migrant women often encounter difficulties in accessing high-quality care. For instance, unfamiliar interventions and practices, such as the use of ultrasounds and electronic fetal monitoring can cause distress and withdrawal from care [9, 10]. Additionally, displacement between or within countries can hinder the continuity of care: in many host countries, asylum seekers are subject to ‘dispersal’ policies and may be moved from one accommodation facility to another several times while their asylum claim is being processed [11]. Women who have recently moved and lack knowledge about the health care system of their host country may not be able to consult the same care team through their pregnancy, labour and postpartum experience [12, 13]. This fragmented course of maternity care can result in difficulties in building trusting relationships with care providers [14]. Experiences of discrimination or unfair treatment and lack of trust towards health care providers can also hinder engagement with maternity care services [15]. These issues may lead to reduced use of maternity care services, particularly antenatal services, by refugees and asylum seekers [15–17] and the diminished use of antenatal health services can result, in turn, in adverse perinatal outcomes [18].

While the literature highlights these complex factors affecting the maternity care experiences of people seeking sanctuary, there is a recognised lack of high-quality disaggregated quantitative data on perinatal clinical interventions among refugees and asylum seekers, and the limited research findings tend to be inconsistent [3, 19, 20]. Indeed, some publications report lower rates of clinical interventions during labour and delivery among refugee women (e.g. planned caesarean section, induction of labour [9]), whereas some report higher rates (e.g. caesarean section [21]), and yet others report no significant differences between refugees and other population groups (e.g. caesarean section [22–24]).

These clinical interventions performed during labour and delivery can reduce adverse outcomes when they are indicated, but unnecessary interventions may affect the process of labour and delivery, trigger a cascade of other interventions, and pose additional risks to the mother and baby [20, 25]. Thus, inadequate perinatal care may consist of insufficient interventions – ‘too little too late’ – or excessive unnecessary interventions – ‘too much too soon’ [26]. The delivery of perinatal healthcare services to different population groups constitutes an important dimension of health equity, and the monitoring of clinical procedure rates, particularly among vulnerable communities, can enhance our understanding of care provision and inform future policy and practice, in alignment with multiple United Nations Sustainable Development Goals (SDG) for 2030: ensuring health and wellbeing, achieving gender equality, and reducing inequality [27].

In the present review, we build upon the existing literature and add to previous reviews by presenting an up-to-date overview of the available evidence regarding the maternity care of refugees and asylum seekers, focusing specifically on quantitative findings comparing intrapartum clinical interventions among refugees and asylum seekers and a non-refugee comparator group in high-income countries, to highlight trends in the data across multiple geographical settings and to identify any disparities in the provision of maternal healthcare services.

### Objectives of the review

Through this scoping review, we aim to systematically document the evidence regarding rates of intrapartum clinical interventions among refugees and asylum seekers in high-income countries (HICs) compared to other population groups. Specifically, our objectives are as follows:

- to identify original research findings regarding the rates of intrapartum clinical interventions among refugees and asylum seekers in high-income countries published since 2011;
- to describe the characteristics of the included publications;
- to report and compare findings regarding rates of intrapartum clinical interventions among refugees and asylum seekers in high-income countries;
- to synthesise findings, identifying gaps in the literature and areas where future research is needed.

## Methods

We conducted a scoping review of the literature, using the methodological guidance provided by the Joanna Briggs Institute guidance for scoping reviews [28], to document the evidence regarding rates of intrapartum clinical interventions among refugee and asylum seekers in high-income countries (HICs) as compared to other population groups. As the available evidence is reported to be limited and inconsistent [19, 20], a scoping review was an appropriate method to use in order to provide an overview of the literature, describe the research being conducted in this area, and identify knowledge gaps [29].

### Information sources and search strategy

We searched four academic databases, Medline, ASSIA, CINAHL and Web of Science, using search terms related to the refugee population, maternity care, and outcomes of interest. Where available, relevant MeSH terms and subject headings were used. While literature reviews were excluded from the present scoping review, the reference lists from published reviews that surfaced during the literature search and those of included publications were manually searched for any additional relevant publications.

### Inclusion criteria

This scoping review includes peer-reviewed studies published in English since 2011 that report original quantitative findings regarding intrapartum clinical interventions (including medical pain relief, labour induction and augmentation, episiotomies, instrumental deliveries, and caesarean sections) among refugees and/or asylum seekers in high-income countries compared to those in any non refugee or non asylum seeker population group.

The operational definitions of refugee and asylum seeker populations can vary widely between research publications as immigration status data are often lacking, incomplete, or inaccessible [30–34]. This scoping review includes research where “refugees” and “asylum seekers” are defined by the authors through a variety of measures, including immigration records or proxy measures such as country of birth.

### Exclusion criteria

Studies where data on refugee and/or asylum seeker populations were not disaggregated, where ‘undocumented migrants’ (including, for example, economic migrants who overstayed their visa) were included in the study group, and where participants were explicitly under the age of 18 (i.e., child or teenage pregnancies) were excluded. Literature reviews reporting no original findings were also excluded.

### Study selection

All the references were uploaded into Endnote and duplicates were removed. We then exported the citations into an Excel spreadsheet to track the screening process. Titles and abstracts were independently screened against the inclusion criteria by two reviewers (Author 1 and Author 2). The two reviewers retrieved all potentially relevant sources in full and assessed them in detail against the inclusion criteria. Any disagreements were discussed within the review team and a third experienced reviewer (Author 3) was involved to reach consensus as needed. We recorded and reported the reasons for the exclusion of papers at the full-text screening stage. The process and results of the search are reported in a PRISMA flow diagram.

### Data collection

We extracted the data using a data extraction tool adapted from the JBI methodology guidance for scoping reviews template. The tool was piloted by two reviewers (Author 1 and Author 2) in a sample of five publications of varied research designs to assess any limitations and improve the tool. The authors of the included publications were contacted as needed to obtain additional information or clarification regarding the published data.

The data extracted included the following:

1. Author(s)
2. Year of publication
3. Study setting
4. Study group and comparison group
5. Key findings related to intrapartum clinical interventions, including count data and effect measures

### Synthesis of findings

The characteristics of the papers included in the review are presented in a table and reported narratively. A meta-analysis of the findings was not feasible due to the heterogeneity of the included studies in terms of setting, population(s), and interventions. In accordance with the guidelines for literature reviews without meta-analysis [35], we chose a standardised metric to compare results, the odds ratio (OR). Unadjusted ORs are reported in forest plots to graphically represent the heterogeneity of results and provide a broad overview of the evidence. Where adjusted ORs are presented and differ significantly from the unadjusted results, we discuss these adjusted results and their implications narratively.

Where ORs were not presented in the publications but count data indicated non-zero group sizes, we transformed the available results into an OR using the number of cases (n) or percentage proportions reported in each publication for each group of interest and the accepted mathematical formula [36].

Many of the included publications present several data points for different study groups; each of these data points are reported separately.

## Results

### Summary of included publications

**Fig. 1:**
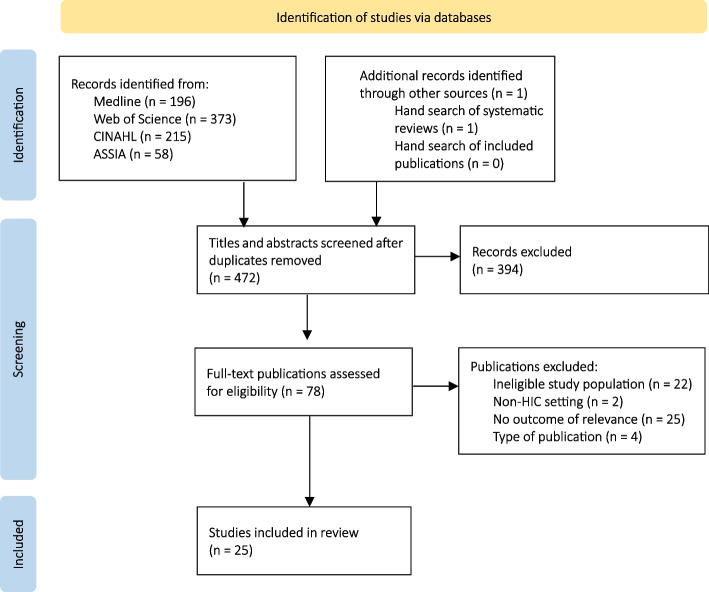
Flowchart of the identification, screening and inclusion of papers

From a total of 472 publications screened for eligibility, 25 publications were included in the review (see Fig. 1). The main characteristics of the included studies are presented in Table 1. Most of the studies were conducted in North America (*n* = 11) [21, 37–46] or Europe (*n* = 7) [23, 47–52], with a further five studies in Australia [9, 53–56] and two in Israel [57, 58].

**Table 1:** Characteristics of the included publications

Author, year (reference)	Country	Study population (n)	Comparison population (n)	Data sources	Outcomes investigated
Aasheim et al., 2020 [47]	Norway	Refugees identified through Statistics Norway migration data (8913)	Norwegian population [Norwegian-born women with Norwegian-born parents] (445 124)	Medical Birth Registry of Norway (MBRN) and Statistics Norway	Pain management (epidural)
Agbemenu et al., 2019 [37]	United States of America (USA)	Refugees identified through country of birth: Burundi, Democratic Republic of Congo, Eritrea, Rwanda, and Somalia (789)	US-born White (56 615); Black women (17 487)	Enhanced electronic birth certificate data extracted from hospitals	Caesarean section (primary, repeat) Induction of labour
Agunwamba et al., 2022 [38]	USA	Refugees identified through country of birth: Somalia (474)	Non refugee women (387)	Rochester Epidemiology Project (REP)	Caesarean section
Ammoura et al., 2021 [48]	Germany	Refugees identified through hospital’s information system (907)	German Federal Obstetric Analysis for the year 2016	Hospital records	Caesarean section Instrumental delivery Episiotomy
Bakken et al., 2015 [49]	Norway	Refugees identified through “conflict-zone” country of birth: Somalia (278), Iraq (166), Afghanistan (71), Kosovo (67)	Norwegian population [born in Norway and had two Norwegian-born parents and four Norwegian-born grandparents] (6826)	Medical Birth Registry of Norway (MBRN) and Statistics Norway	Caesarean section (all, emergency, elective) Induction of labour Instrumental delivery Pain management (epidural analgesia)
Biro and East, 2017 [53]	Australia	Refugees identified through country of birth: countries from which two thirds or more of immigrants entered via Australia’s humanitarian program (1547)	Women of non refugee background (18 020)	Birthing Outcome System, Commonwealth Settlement Reporting Facility data	Caesarean section (elective, emergency) Induction of labour Instrumental delivery
Bozorgmehr et al., 2018 [50]	Germany	Asylum seekers identified through a recorded cost unit associated with state-mandate (569)	Resident women (19 295)	Hospital records	Caesarean section
Clarfield et al., 2023 [39]	Canada	Refugees identified through use of the Interim Federal Health Program (IFHP) (196)	Non refugee patients (13 734)	Hospital records and Ontario Perinatal Record	Caesarean section Instrumental delivery Induction of labour
Correa-Velez and Ryan, 2012 [9]	Australia	Refugees identified through country of birth: African countries (83)	Broader population who gave birth at Mater Mothers’ Hospital in 2007 (3821)	Hospital records	Caesarean section (all, elective) Induction of labour Instrumental delivery (forceps, vacuum) Episiotomy
Gagnon et al., 2013a [41]	Canada	Refugees identified through interviewer-assisted eligibility questionnaires (149)	Non refugee immigrants (505)	Medical records and interviewer-assisted questionnaires	Caesarean section (emergency)
Gagnon et al., 2013b [40]	Canada	Refugees identified through self-declared status, health insurance status, and period living in Canada (72)	Canadian-born women (2482)	NORMAPERS study database	Caesarean section
Gibson-Helm et al., 2014 [54]	Australia	Refugees identified through country of birth: countries from which two thirds or more of the total immigrants had entered Australia within the humanitarian stream – North Africa (1147), Middle and East Africa (87) and West Africa (45)	Non refugee immigrants from North Africa (214), Middle and East Africa (619) and West Africa (61)	Birthing Outcomes System	Caesarean section Instrumental delivery Induction of labour Pain management (analgesia)
Gibson-Helm et al., 2015a [55]	Australia	Refugees identified through country of birth: countries from which at least two-thirds of the total immigrants entered Australia through the Humanitarian Program—South Asia (1930), Southeast Asia (107) or West Asia (287)	Non refugee immigrants from South Asia (7412), Southeast Asia (5574) or West Asia (990)	Birthing Outcomes System	Induction of labour Caesarean section (all, nulliparous term singleton vertex) Instrumental delivery Episiotomy
Gibson-Helm et al., 2015b [56]	Australia	Refugees identified through country of birth: countries from which 67–100 percent of immigrants entered Australia within the humanitarian migration stream and < 10 percent entered within the skilled migration stream (2713)	Non refugee immigrants (10 606)	Birthing Outcomes System	Caesarean section Induction of labour Instrumental delivery Pain management (analgesia) Episiotomy
Gil et al., 2020 [57]	Israel	Refugees identified through country of birth: Eritrea and Sudan (635)	Israeli women (1270)	Electronic medical records	Caesarean section Induction of labour Instrumental delivery Episiotomy Pain management (epidural analgesia)
Goble et al., 2023 [42]	USA	Refugees identified through country of birth: Democratic Republic of the Congo and Somalia (289)	US-born women (4635) and non refugee migrants (1074)	Hospital records	Caesarean section Induction of labour Instrumental delivery Pain management (maternal analgesia, epidural anaesthesia, spinal anaesthesia)
Kandasamy et al., 2014 [21]	Canada	Refugees identified through use of the Interim Federal Health Program (IFHP) (274)	Non refugee controls (273)	Hospital records	Caesarean section
Kentoffio et al., 2016 [43]	USA	Refugees identified through use of a refugee clinic (53 individuals – 116 pregnancies)	Spanish-speaking non refugee migrants (186 – 368 pregnancies); English-speaking controls (136 – 279 pregnancies)	Electronic health records	Caesarean section Instrumental delivery
Khan et al., 2017 [44]	Canada	Refugees with Gestational Diabetes Mellitus, migration status identified through the Permanent Resident Database of Immigration, Refugees and Citizenship Canada (PRD-IRCC) (2106)	Non refugee migrants with Gestational Diabetes Mellitus (16 232); Non migrants with Gestational Diabetes Mellitus (22 564)	Hospital discharge data, Physician service claims data, Registered Persons Database, Ontario Diabetes Database, Permanent Resident Database	Caesarean section
Michaan et al., 2014 [58]	Israel	Refugees identified through country of birth: Eritrea and Sudan (247)	Native Israelis (247)	Hospital records	Caesarean section Pain management (epidural analgesia) Episiotomy
Sturrock et al., 2021 [51]	United Kingdom (UK)	Asylum seekers identified through residence at a hostel for people seeking asylum (34)	Non-asylum-seeking women (68)	Badgernet electronic patient record system, electronic patient records	Caesarean section (elective, emergency) Instrumental delivery (forceps, ventouse)
Van Hanegem et al., 2011 [23]	Netherlands	Asylum seekers with severe acute maternal morbidity, migration status identified through web-based forms filled by hospital staff for the LEMMoN study (40)	Total Dutch pregnant population, excluding asylum seekers (37 021); Dutch women with severe acute maternal morbidity (2512); Non-Western immigrants, excluding asylum seekers (517)	LEMMoN study records and Statistics Netherlands	Caesarean section Induction of labour
Van Zandt et al., 2016 [45]	USA	Refugees identified by birth companions (144)	Non refugees	Data collected by program birth companions	Caesarean delivery Induction of labour (Pitocin induction or augmentation) Pain management (epidural use)
Verschuuren et al., 2020 [52]	Netherlands	Asylum seekers identified through residence in asylum seeker centres (344)	Dutch population (2323)	Combined databases of Primary and secondary care practices	Caesarean section (all, primary) Induction of labour Instrumental delivery Pain management (opioid analgesic, epidural)
Wanigaratne et al., 2018 [46]	Canada	Refugees identified through the Immigration and Refugees Citizenship Canada Permanent Resident Database (IRCC-PRD): all births (52 360) and first births (29 023)	Canadian-born population (977 045); Non refugee migrants (29 023)	IRCC-PRD, Discharge Abstract Database, Office of the Registrar General’s Vital Statistics-Death registry, Ontario HIV Database	Caesarean section

Across these publications, researchers have used various operational definitions of the study population to identify refugees and/or asylum seekers. Indeed, while several authors have used immigration status as recorded in administrative data, many others have used indirect measures of immigration status such as the utilisation of specific programmes or services (e.g. refugee clinics, accommodation centres, health insurance programmes) or proxy measures such as country of birth.

The mode of delivery is by far the most reported intrapartum clinical intervention in the literature, with all but one of the included papers presenting rates of caesarean sections and 12 publications reporting rates of instrumental vaginal deliveries. Induction or augmentation of labour was reported in nearly half of the publications (*n* = 12), whereas the use of analgesia during labour and episiotomies were reported in eight and seven papers, respectively. The latter types of clinical interventions thus appear to be understudied in the literature pertaining to the maternity care of refugees and asylum seekers in high-income countries as compared to mode of delivery.

## Summary of results

Overall, the reported results often have very wide confidence intervals, indicating that the results have a low level of precision, and several of the confidence intervals overlap with the null value, indicating that the reported difference in the odds of intrapartum clinical interventions between the study group and the comparison group may be due to chance. These inconclusive findings represent areas needing additional research, particularly to achieve a higher degree of statistical precision and detect potential differences between groups more accurately. The findings reported in the included studies for each type of perinatal intervention are presented in the following sections.

### Induction and augmentation of labour

**Fig. 2:**
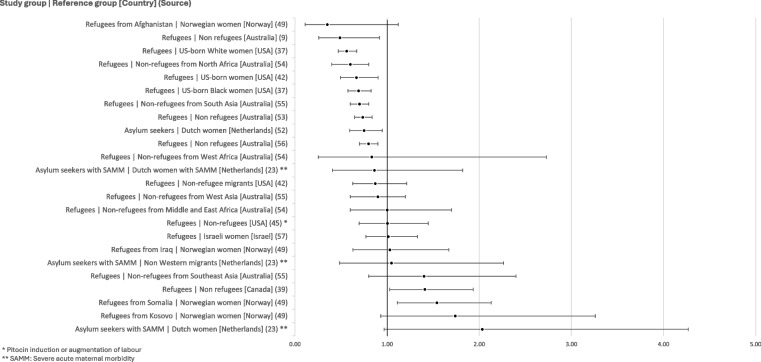
Unadjusted odds ratios for rates of induction or augmentation of labour

The reported results for rates of induction and augmentation of labour tend to be inconsistent. Most of the unadjusted ORs (*n* = 13) overlap with the null value, indicating that there may be no significant difference in the odds of induction or augmentation of labour between the study groups and comparison groups. However, nine analyses indicate significantly lower odds of induction or augmentation of labour among refugees or asylum seekers, while two indicate significantly high odds in the study groups (see Fig. 2).

Four publications presented multivariable regression analysis models to examine the effect of possible confounders. The large majority of models presented in the four publications [49, 54–56] present no significant differences between any of the groups of refugees and asylum seekers and comparator groups, indicating that there may be no independent association between refugee or asylum seeker status and the induction or augmentation of labour.

However, adjusting for maternal age and parity, Bakken et al. [49] show significantly greater odds of induction for refugees from Somalia (adjusted OR: 1,92 CI: 1,3–2,7) and from Kosovo (adjusted OR: 2.23 CI: 1.18–4.23) compared to Norwegians. Adjusting for maternal age, parity, body mass index (BMI) and a measure of socioeconomic disadvantage, an Australian study shows that refugees from Southeast Asian countries of birth have greater odds of induction of labour than non refugee immigrants from the same region (adjusted OR: 2.0 CI: 1.1–3.5), while South Asian refugees appear to have lower odds than non refugee immigrants from the same region (adjusted OR: 0.9 CI: 0.8–1.0) [55]. Interestingly, Gibson-Helm et al. [56] report a null value for the odds ratio – adjusted for maternal age, parity, BMI and a measure of socioeconomic disadvantage – between refugees from all humanitarian source countries (i.e. countries from which over two thirds of immigrants entered Australia within the humanitarian migration stream and under ten percent entered within the skilled migration stream) and immigrants from nonhumanitarian source countries (adjusted OR: 1.0 CI: 0.9–1.1), which could suggest that varying patterns across different subgroups of refugees may muddle results in larger, heterogeneous study groups.

### Analgesia

**Fig. 3:**
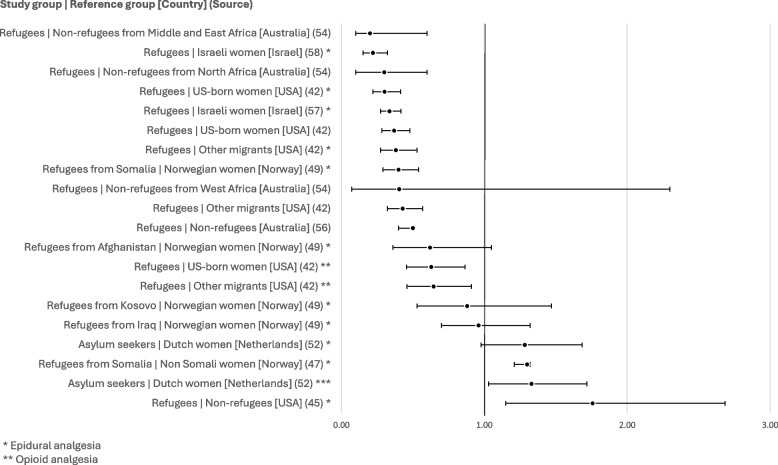
Unadjusted odds ratios for rates of analgesia utilisation

A total of eight publications present rates of clinical analgesia, either as a general category (pain relief), or for a specific method of analgesia (epidural analgesia or the use of opioids). While several unadjusted odds ratios overlap the null value, indicating the possibility of no significant difference between groups, most unadjusted ORs seem to indicate lower odds of clinical pain relief among refugee and asylum seeker populations (see Fig. 3).

Several authors investigated the effect of potential confounding factors on the link between refugee or asylum seeker status and the utilisation of analgesia in labour through multivariable analysis. Aasheim et al. [47], for instance, show that adjusting results for year of birth, health region, maternal age, marital status, education, size of hospital, and gross income decreases the unadjusted OR of 1.3 (1.21–1.32) to 0.83 (0.79–0.87). Similarly, Bakken et al. [49] produced several multiple logistic regression analyses for each of their four study subgroups. While most of the models presented do not show notable differences between the unadjusted and adjusted results, confounders seem to influence the relationship between the incidence of epidural analgesia and refugee status among refugees from Kosovo compared to Norwegians: adjusting for maternal age, parity, marital status, and educational level reduces the OR to 0.48 (0.27–0.84). Adjusting for obstetric and maternal factors in addition to the previously listed variables results in an almost identical OR (0.48 [0.27–0.85]). These results echo the majority of reported unadjusted findings, suggesting that odds of analgesia during labour appear to be lower among refugees than among individuals in other populations, even when considering other predictor variables.

### Caesarean section

**Fig. 4:**
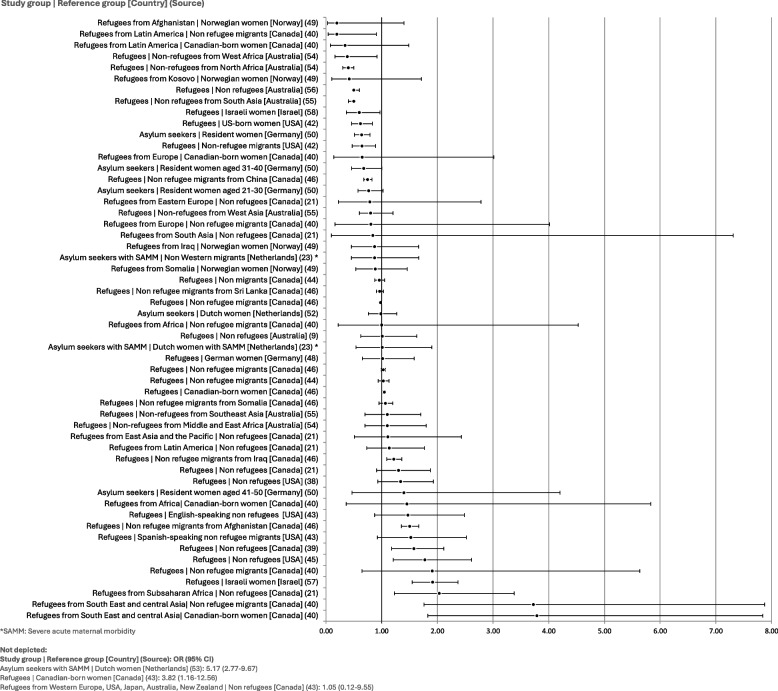
Unadjusted odds ratios for rates of caesarean section

Caesarean section rates are by far the most reported intervention rates in the included publications, with all but one article reporting on caesarean sections. A broad overview of the unadjusted ORs (see Fig. 4) indicates inconsistent findings: 27 data points indicate lower odds of caesarean section among refugees and asylum seekers than among other populations (ten of which are statistically significant, with the confidence interval not overlapping the null value), while 29 data points (18 of which are statistically significant) indicate the opposite, and one publication presents an OR of exactly 1.00, suggesting no difference between groups at all [40].

Several multivariate analyses demonstrate that confounders affect the relationship between caesarean section rate and refugee status. After adjusting for age, education, BMI, parity, and gestational hypertension, Agunwamba et al. [38] obtain a greater OR when comparing Somali refugees to non-Somali women (1.81 [1.15–2.84]). Additionally, Bozorgmehr et al. [50] demonstrate that other predictor variables lessen the statistical significance of the relationship between asylum-seeker status and caesarean section rates: the unadjusted OR of 0.64 (CI: 0.51–0.79) increases to 0.84 (CI: 0.66–1.07) when adjusting for age, length of admission and high-risk conditions.

**Fig. 5:**
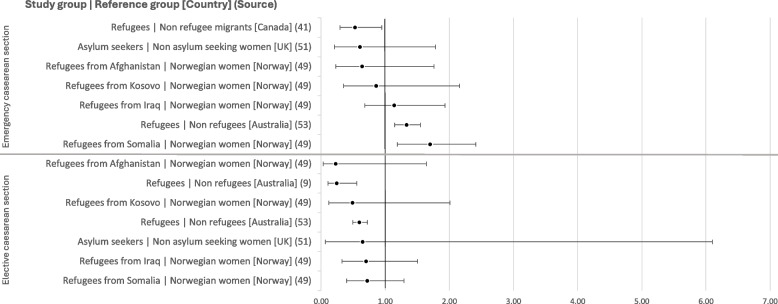
Unadjusted odds ratios for rates of emergency and elective caesarean section

Analyses of different types of caesarean sections or subgroups of refugees and asylum seekers may shed light on the variability of the results. Indeed, while analyses of elective caesarean section all show lower odds among refugee populations than among other groups, findings differ in regard to emergency caesarean sections, with two studies showing significantly greater odds of emergency caesarean sections among three groups of refugees than among other populations [49, 53] (see Fig. 5). Furthermore, Clarfield et al. found significantly greater odds of emergency caesarean section among refugees when adjusting for maternal age, pre-existing hypertension, and hypertension of pre-eclampsia in the current pregnancy (adjusted OR: 1.08 [1.00–1.17], 35.18 [3.59–344.73], and 5.69 [1.25–25.85], respectively), whereas adjusting for the number of living children resulted in lower odds of emergency caesarean section among refugees (adjusted OR: 0.27, CI: 0.11–0.67). Gagnon et al. [41] also found that refugee status was linked to lower odds of emergency caesarean section as compared with other migrants when adjusting for several confounders linked to migration, social indicators, health services, and biomedical indicators (0.45 [0.20–0.99]).

**Fig. 6:**
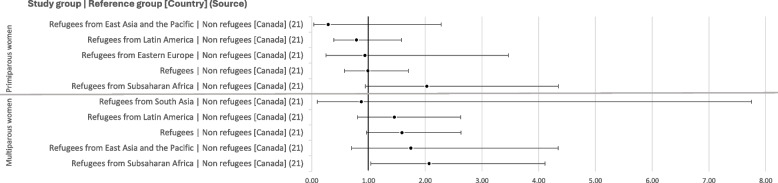
Unadjusted odds ratios for rates of caesarean section among primiparous and multiparous women

Additionally, disaggregating the data for primiparous and multiparous women reveals some diverging trends between the two groups. Kandasamy et al. [21] show that multiparous refugees tend to have greater odds of having a caesarean section than non refugees (1,59 [0.96–2.63]), while primiparous refugees have similar or slightly lower odds (0.99 [0.57–1.71]). This phenomenon is particularly evident for refugees from East Asia and the Pacific (1.75 [0.70–4.34] vs 0.29 [0.04–2.28]) and those from Latin America and the Caribbean (1.46 [0.81–2.62] vs 0.79 [0.39–1,58]) compared to non refugees (see Fig. 6).

Echoing these results, Gibson-Helm et al. [55] show low ORs in their analysis of caesarean sections among low-risk first-time refugee mothers (nulliparous term singleton vertex caesareans) as compared to non refugees from South Asia, Southeast Asia and West Asia (0.71 [0.57–0.89], 0.54 [0.19–1.56], and 0.45 [0.20–1.03] respectively). In the case of refugees and non refugees from West Asia, adjusting for age, parity, BMI and a measure of socio-economic status reduces the OR to 0.3 (CI: 0.1–0.9) [55].

### Instrumental vaginal delivery

**Fig. 7:**
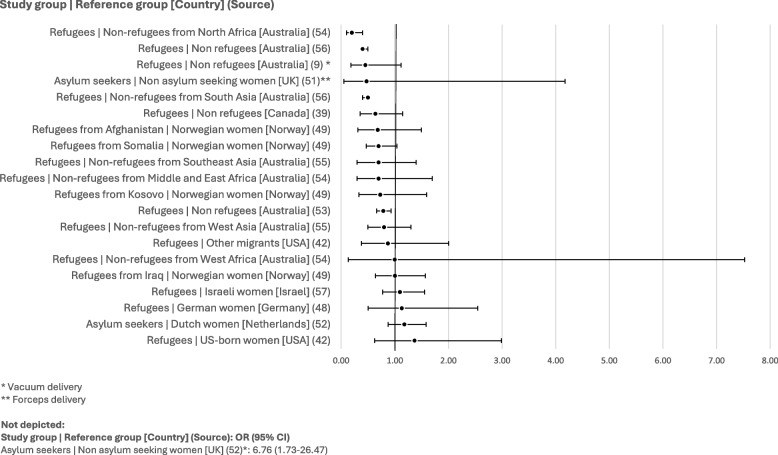
Unadjusted odds ratios for rates of instrumental vaginal delivery

Nearly all the unadjusted ORs indicate lower odds of instrumental vaginal delivery among refugees than among individuals in other populations (see Fig. 7). However, some studies examining a specific type of instrumental delivery show diverging results: in a small study of 34 asylum seekers and 64 controls, Sturrock, Williams [51] show non-significantly lower odds of forceps delivery (0.48 [0.05–4.18], but greater odds of ventouse delivery (6.76 [1.73-26.47]) among asylum seekers than those in the control group. Correa-Velez and Ryan [9], however, report non-significantly lower odds of vacuum delivery among refugees than among the broader population (0.45 [0.18-1.12]).

However, while some multivariable analyses show little difference from the unadjusted results, adjusting for maternal age, parity, BMI and a measure of socioeconomic status raised the OR to 1.00 (0.3–2.8) for refugees compared to non refugees from Middle and East Africa [54], indicating that confounders may affect the above results.

### Episiotomy

**Fig. 8:**
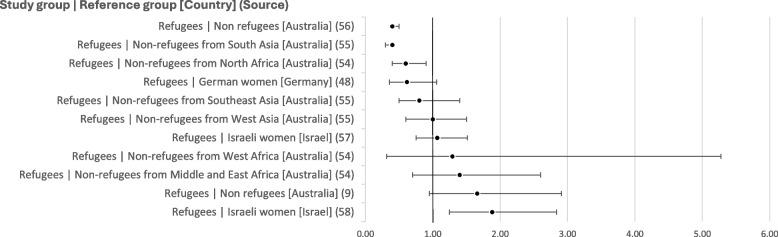
Unadjusted odds ratios for rates of episiotomy

The unadjusted ORs indicate inconsistent findings regarding episiotomy rates: five data points indicate lower odds of episiotomy among refugees than among other populations (three of which are statistically significant with a confidence interval that does not overlap the null value) and five data points indicate the opposite trend (with one result being statistically significant), with one additional data point indicating an OR of exactly 1.00 [55] (see Fig. 8). Adjusting for maternal age, parity, BMI and a measure of socio-economic status raises the OR for one subgroup, reaching 1.0 (0.5 to 1.8) for refugees compared to non refugees from North Africa [54].

## Discussion

The inconsistency of findings, not only between papers but also between different subgroups within the same study, highlights the fact that refugees and asylum seekers do not constitute a homogenous group and suggests that complex interrelated factors could be at play. Overall, the results reported in the reviewed publications are imprecise and cannot be generalised to the wider asylum-seeking population or directly compared between one another as researchers have used different methodologies, conducting their research in distinct national or regional contexts, and targeting specific population groups. Due to the heterogeneity of the included studies, we must exercise caution in the interpretation of the results of this review, avoiding direct comparisons and generalisation of findings. However, this scoping review provides a comprehensive overview of the broad body of literature concerning the maternity care of refugees and asylum seekers.

While findings related to the mode of delivery, particularly rates of caesarean section, were broadly reported in the literature, fewer publications included results concerning induction or augmentation of labour, clinical analgesia, and episiotomies. Additional research into the rates of these underrepresented clinical interventions could provide additional insight into potential inequities in the provision of maternity care for different population groups.

The findings reported in this review reveal some inconsistent trends in intrapartum clinical interventions among refugees and asylum seekers in high-income countries, although some patterns are clearer. The results tend to indicate lower odds of clinical pain relief utilisation among refugees and asylum seekers which is coherent with the literature showing racial and ethnic disparities in pain management in general [59, 60] and specifically in peripartum care [61, 62]. These disparities could be linked to differences in individual pain management preferences and expectations such as the desire for an unmedicated birth, communication barriers between patients and care providers [61, 62], differences in physiological response to pain, and inequities or discrimination in clinician decision making, such as withholding or delaying the provision of pain relief to some patients [59]. The use of epidural analgesia in labour is, in turn, associated with higher rates of obstetric interventions including instrumental vaginal deliveries [63, 64]. While the exact causal link between epidural analgesia and further clinical interventions remains unclear, it could be associated, for example, with the motor block or motor weakness that can be caused by epidurals [64]. The findings reported in this review showing lower rates of instrumental delivery among refugees and asylum seeker are thus consistent with the literature: lower rates of clinical analgesia among this group, due to multiple interrelated factors, could contribute to a reduced need for instrumental delivery.

No consistent trend is found regarding rates of induction of labour and episiotomies: several studies showed no statistically significant difference between refugees and asylum seekers and the comparator groups, while some reported higher odds and yet others reported lower odds of these interventions among the study group. While caesarean section rates are the most commonly reported rates in the included publications, the findings reported in the literature tend to diverge.

In several of the included publications, the authors present the results of multivariable analysis models to explore the effects of several potential confounders, including maternal age, parity, measures of socioeconomic status, BMI, and the presence of health conditions. The wide range of confounders examined in the literature, from physiological characteristics to socioeconomic status, illustrates the complexity of the relationship between immigration and health. Some of the multivariable analyses presented in the included studies resulted in similar findings to the univariable analyses, suggesting very small or no confounding effects on the outcome of interest. However, several multivariable models showed that confounders can have a clear effect on the relationship between refugee or asylum seeker status and some outcomes. In a particularly stark example, Aasheim et al. [47] report a crude OR of 1.3 (95% CI: 1.21–1.32) for epidural analgesia among refugees in reference to non-migrants and an adjusted OR of 0.83 (95% CI: 0.79–0.87) when adjusting for maternal age, marital status, education, gross income, year of birth, size of hospital and health region. These results suggest that while refugees initially appear more likely to receive analgesia, adjusting for known confounders revealed the opposite association between refugee status and analgesia. The results presented in the included publications illustrate the importance of conducting multivariable analyses to establish the direction and strength of the relationship between immigration status and maternal health outcomes while considering the effects of complex confounders.

### Strengths and limitations

This scoping review allows for a broad overview of the available evidence concerning refugees’ and asylum seekers’ experiences of maternity care in high-income countries and highlights gaps in the literature, including the need for high-quality disaggregated data to investigate potential patterns, particularly regarding the induction and augmentation of labour, caesarean section, and episiotomy rates. However, as only English language publications were included, relevant research produced in languages other than English was not identified. Additionally, all the included studies originated from a total of only nine countries, representing a small proportion of high-income countries.

The heterogeneity of the included studies regarding the study setting, study group, and specific intervention limits the comparability of the results. Furthermore, the use of indirect or proxy measures to identify the study group in many of the included studies signifies that the reported findings may not be generalizable to people with refugee or asylum seeker status.

As a scoping review, no quality appraisal was undertaken of studies included in the review which limits interpretation, as inadequately designed or poorly conducted studies may be affected by biases and should be interpreted with caution.

### Future directions

While some multivariable analyses indicate that other predictor variables may have a stronger effect on intrapartum interventions, many analyses have demonstrated that the link between refugee or asylum seeker status and various intrapartum interventions persists when controlling for other variables. Further research is needed to better understand the provision of intrapartum care to refugee and asylum-seeking women in high-income countries. Some types of intrapartum clinical interventions, including the provision of analgesia, induction or augmentation of labour, and episiotomies, remain understudied. Examining specific subsets of interventions (e.g., elective and emergency caesareans) or specific subgroups of the population (e.g., primiparous and multiparous women) could shed light on otherwise inconsistent trends. Additional research in countries other than Australia, Canada, and the USA, where a substantial amount of research has already been conducted, would be particularly valuable to facilitate international comparisons and provide a broader overview of the phenomenon. Disaggregated data could provide more detailed insight into this issue, taking into account the heterogeneity and diversity of refugee and asylum seeker populations, institutional and societal contexts, and individual clinical profiles. Systematic, secure, and safe collection of migration data in the context of health research would enhance research capacity. Additionally, further analysis of clinical data, including retrospective chart reviews, in relation to official standards of care, could provide insight into the appropriateness of the clinical interventions performed (or not) in each case. In complement to further quantitative data analysis, qualitative research can provide insight into the varied lived experiences of refugee and asylum-seeking women giving birth and interacting with maternity care services.

## Conclusions

This scoping review provides a broad overview of the available evidence concerning rates of intrapartum clinical interventions among refugees and asylum seekers in high-income countries. The reviewed publications are heterogeneous in scope and methodological design, limiting the comparability of the data. While results are generally imprecise and inconsistent, the data indicate lower odds of clinical pain relief and instrumental delivery among refugees and asylum seekers than among individuals in other population groups. Additional research into subgroups of refugees and asylum seekers, potential confounders, and varied institutional and national settings is needed to further our understanding of the links between immigration status and maternity care, ultimately informing policy and practice to improve perinatal health and the provision of care for all.

## Data Availability

No datasets were generated or analysed during the current study.

## Abbreviations

BMI: Body mass index
HIC: High-income country
OR: Odds ratio
SDG: Sustainable Development Goals
USA: United States of America
